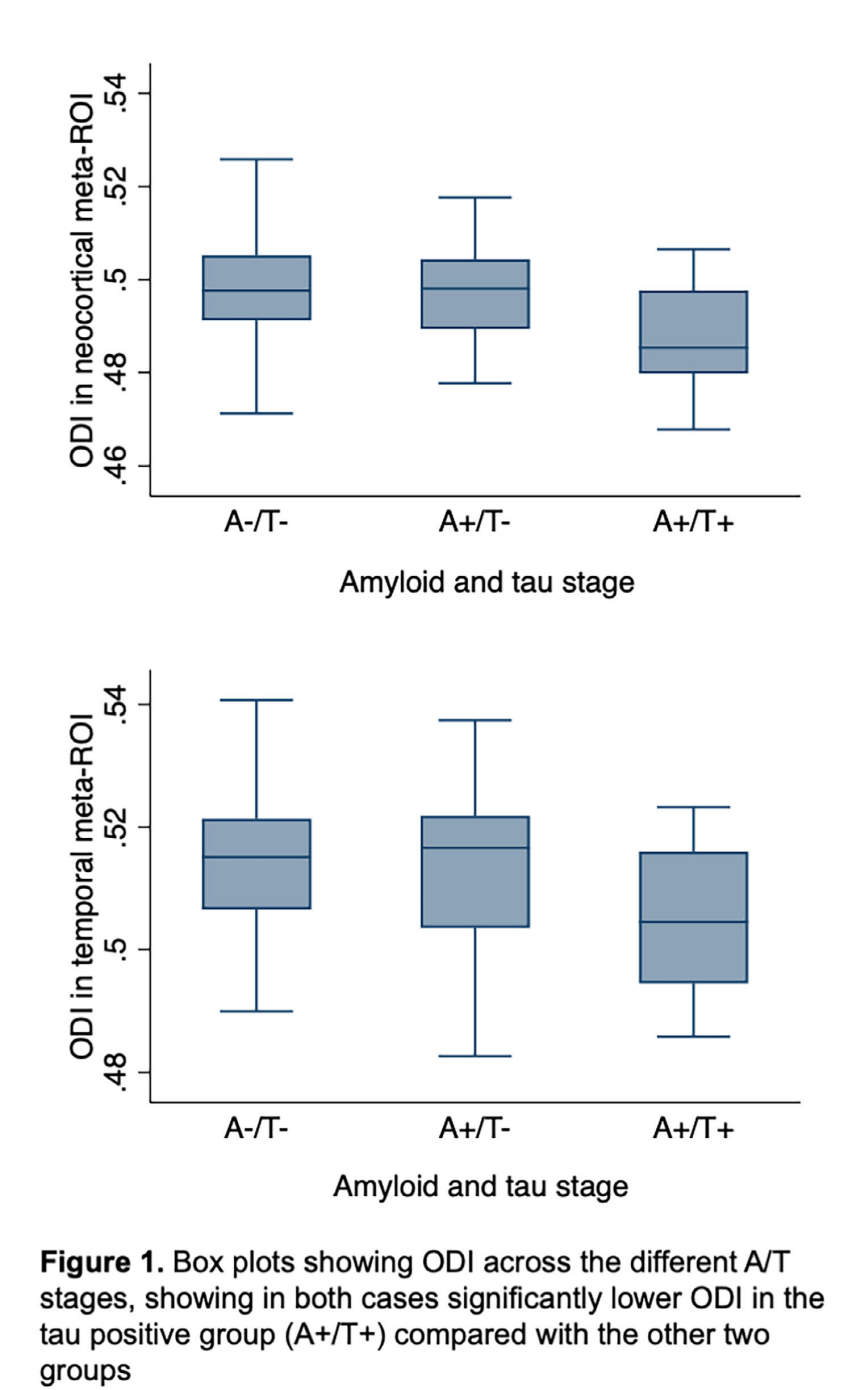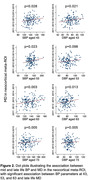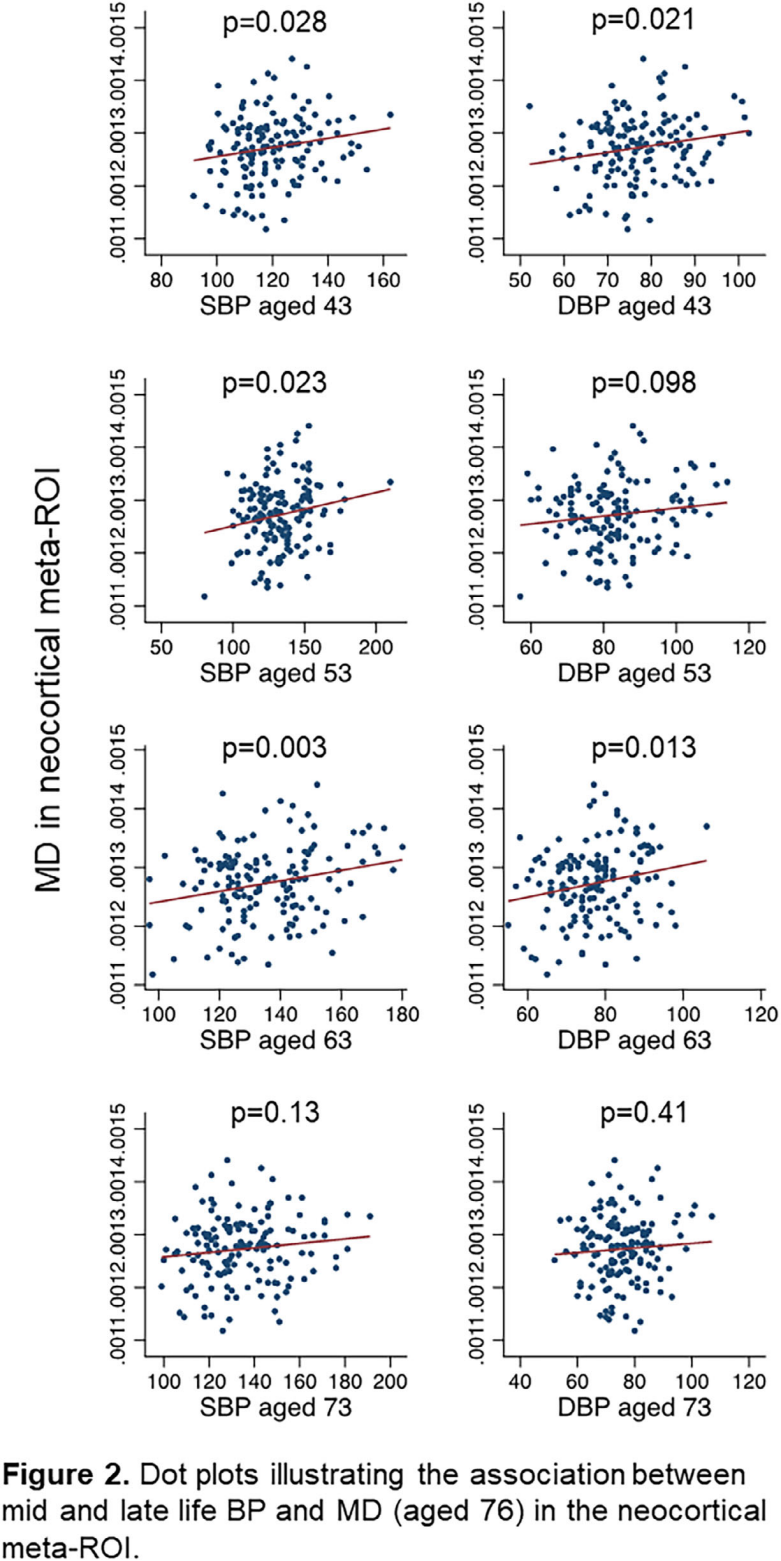# The association between grey matter microstructural MRI biomarkers, mid‐life blood pressure, and late‐life Alzheimer’s disease pathology

**DOI:** 10.1002/alz.092523

**Published:** 2025-01-09

**Authors:** Philip SJ Weston, William Coath, Thomas M. Brown, Catherine J Scott, Ian B. Malone, Erik Arstad, Ramla Awais, Kerstin Sander, David L Thomas, John C Dickson, Michael Schöll, Marcus Richards, Nick C Fox, Hui Zhang, David M Cash, Jonathan M Schott

**Affiliations:** ^1^ UK Dementia Research Institute, London UK; ^2^ Dementia Research Centre, UCL Queen Square Institute of Neurology, London UK; ^3^ Dementia Research Centre, UCL Queen Square Institute of Neurology, University College London, London UK; ^4^ Institute of Nuclear Medicine, University College London Hospitals, London UK; ^5^ Radiopharmaceutical Chemistry, University College London, London UK; ^6^ Neuroradiological Academic Unit, Department of Brain Repair and Rehabilitation, UCL Queen Square Institute of Neurology, University College London, London UK; ^7^ Dementia Research Centre, Department of Neurodegenerative Disease, UCL Queen Square Institute of Neurology, University College London, London UK; ^8^ Wallenberg Centre for Molecular and Translational Medicine, University of Gothenburg, Gothenburg Sweden; ^9^ MRC Unit for Lifelong Health & Ageing at UCL, London UK; ^10^ UK Dementia Research Institute, Queen Square Institute of Neurology, University College London, London UK; ^11^ Centre for Medical Image Computing, UCL, London UK

## Abstract

**Background:**

With an aging population, it is essential to identify subtle features of brain pathology – both neurodegenerative and vascular – at an early stage, which may predict risk of future decline. We used diffusion MRI (dMRI) to assess grey matter cortical microstructure and investigate associations with 1) Alzheimer’s disease (AD) pathology and 2) mid/late‐life vascular risk (as measured by blood pressure (BP)).

**Method:**

151 asymptomatic individuals from the British 1946 birth cohort underwent combined PET/MR with [18F]florbetapir Aβ‐PET at ∼73yrs, and [18F]MK‐6240 tau‐PET at ∼76yrs. Multi‐shell diffusion MRI was acquired; neurite orientation dispersion and density imaging (NODDI) quantified orientation dispersion index (ODI), a proxy measure of dendritic morphology/complexity, with DTI measuring mean diffusivity (MD), a less specific measure of degenerative change. Cortical volume was estimated using GIF. PET SUVRs were calculated. Amyloid load was assessed in a cortical composite region, with tau PET and MRI measures assessed in 1) a temporal meta‐ROI (analogous to early Braak stages), and 2) a larger neocortical meta‐ROI, analogous to later Braak stages. BP measurements were taken throughout mid and late life, at ages 43, 53, 63, and 73.

**Result:**

Seventy participants were amyloid positive (A+), of whom 27 were tau positive (T+). Global cortical ODI was significantly lower in A+/T+ than A+T‐ (p=0.005) and in A+/T+ than A‐/T‐ (p=0.001), but with no significant difference between A+/T‐ and A‐/T‐. The same pattern was found for ODI in the temporal ROI. No consistent differences were found across A/T groupings for MD or volume. MD (both global and temporal) at aged 76 showed significant associations (p<0.05) with BP at ages 43, 53 and 63, but not at 73. These association remained after adjusting for A/T pathology and cortical volume. For ODI, associations with mid‐life BP were less consistent.

**Conclusion:**

Mid‐life BP is associated with late life cortical microstructural breakdown, as measured by MD, in the absence of detectable volume changes and independent of Alzheimer’s disease (AD) pathology. ODI appears to be more sensitive and specific to dendritic tau‐related changes. Cortical dMRI offers promise in the presymptomatic identification, staging and risk stratification of both AD and vascular dementia.